# Multifunctional Magnetic Nanowires: Design, Fabrication, and Future Prospects as Cancer Therapeutics

**DOI:** 10.3390/cancers11121956

**Published:** 2019-12-06

**Authors:** Abu Bakr A. Nana, Thashree Marimuthu, Pierre P. D. Kondiah, Yahya E. Choonara, Lisa C. Du Toit, Viness Pillay

**Affiliations:** Wits Advanced Drug Delivery Platform Research Unit, Department of Pharmacy and Pharmacology, School of Therapeutic Sciences, University of the Witwatersrand, Johannesburg, 7 York Road, Parktown 2193, South Africa; abubakr.nana@gmail.com (A.B.A.N.); thashree.marimuthu@wits.ac.za (T.M.); pierre.kondiah@wits.ac.za (P.P.D.K.); yahya.choonara@wits.ac.za (Y.E.C.); lisa.dutoit1@wits.ac.za (L.C.D.T.)

**Keywords:** magnetic nanowires, cancer, magnetic hyperthermia, magnetic actuation, magnetic drug targeting

## Abstract

Traditional cancer therapeutics are limited by factors such as multi-drug resistance and a plethora of adverse effect. These limitations need to be overcome for the progression of cancer treatment. In order to overcome these limitations, multifunctional nanosystems have recently been introduced into the market. The employment of multifunctional nanosystems provide for the enhancement of treatment efficacy and therapeutic effect as well as a decrease in drug toxicity. However, in addition to these effects, magnetic nanowires bring specific advantages over traditional nanoparticles in multifunctional systems in terms of the formulation and application into a therapeutic system. The most significant of which is its larger surface area, larger net magnetic moment compared to nanoparticles, and interaction under a magnetic field. This results in magnetic nanowires producing a greater drug delivery and therapeutic platform with specific regard to magnetic drug targeting, magnetic hyperthermia, and magnetic actuation. This, in turn, increases the potential of magnetic nanowires for decreasing adverse effects and improving patient therapeutic outcomes. This review focuses on the design, fabrication, and future potential of multifunctional magnetic nanowire systems with the emphasis on improving patient chemotherapeutic outcomes.

## 1. Introduction

Cancer is amongst the most pernicious diseases known, due to the high mortality and incidence rates reported [1]. Traditional treatment such as chemotherapy, radiation therapy, and surgery has provided successful treatment and control of cancer to a certain extent [2]. However, each approach comes with its own difficulties, notably being invasive or unspecific in its killing effect leading to grave adverse effects. Alternative therapies such as carbon ion therapy and proton therapy are more specific and carries a lower side effect risk when compared to X-ray radiotherapy. The limitation of these therapies is that they require specialized equipment and personnel, thus resulting in high cost and constrained treatment accessibility [3]. Chemotherapy, when looked at in isolation, has further shortcomings such as short half-life, acquired drug resistance, nonspecific bio-distribution in cells and tissues, rapid metabolism, and excretion. This leads to a low therapeutic index due to the destruction of healthy cells and potent toxicity [1].

**Figure 1:**
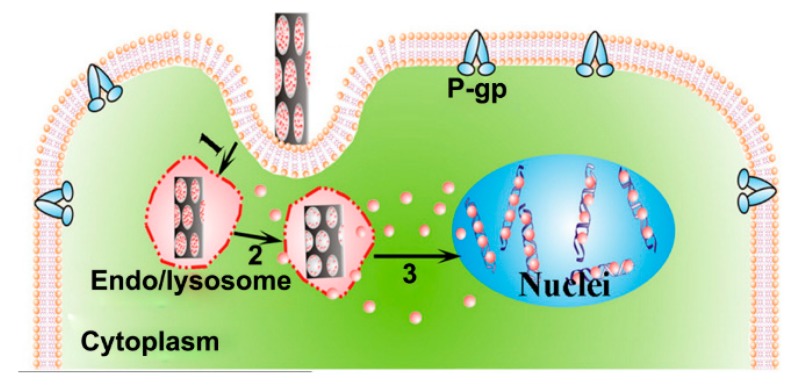
Schematic showing doxorubicin loaded nanowires being internalized into the cytoplasm and releasing the drug from the pH stimuli from the endo/lysosome. Where P-gp is P-glycoprotein. Adapted with permission from Peng et al. [4].

Multifunctional nanosystems has been of recent interest in anti-cancer-therapy for the purpose of developing safe, effective, and efficacious drug delivery systems, due to the potential of overcoming the disadvantages of traditional strategies. Amongst the most promising multifunctional nanosystems include the use of magnetic drug targeting, actuation, or hyperthermia in amalgamation with triggered drug release strategies as well as their combination with diagnostic methods such as magnetic resonance imaging and fluorescence imaging. This can lead to a theranostic approach personalizing cancer treatment for patients. Amongst the various nanoparticles and nanocarriers that multifunctional nanosystems are comprised of, nanowires (NWs) are of key interest due to their shape anisotropy and large surface area. Figure 1 shows a NW system, in which stimuli release the drug in the presence of a decreased pH after cellular internalization.

NWs have widespread applications in various fields including drug delivery [5], sensors [6], biomedicine [7], water purification [8], magnetic storage [9], and electronics [10]. NW application in drug delivery includes the use in both targeted drug delivery systems such as magnetically responsive platforms [11,12] and triggered release systems such as pH responsive systems [13]. NWs are also used to induce non-chemotoxic cell death by using magnetic actuation and induced localized hyperthermia in the presence of an alternating magnetic field.

NW are structurally characterized as one-dimensional geometry, involving large lengths reaching micrometer range and small diameters in the nano-range (~10–200 nm). Their length-to-diameter ratio (aspect ratio) is usually large [14], which differentiates them from nanorods.

These intrinsic properties of NW provide specific advantages in terms of drug delivery, which is of particular interest with regards to cancer therapeutics, such as a large surface-area-to-volume ratio and increased biocompatibility by its ability to camouflage and be coated with various biocompatible and biodegradable coatings (biopolymers and semi-synthetic polymers) increasing its solubility, stability, and its ability to be functionalized. NWs therefore provide an efficient platform for drug delivery systems to be based on. The large surface-area-to-volume ratio allows for greater drug loading and attachment of targeting molecules while the small diameters provide the ability to pass through narrow capillaries [15]. With regards to magnetically responsive NW, the elongated shape brings inherent advantages that can be exploited. Their anisotropic magnetic and physical properties allow for easy magnetization, greater magnetic moments when compared to spherical particles, and NWs also have large remnant magnetization. The large remnant magnetization intensifies the effectiveness and range of the magnetic interactions due to its favorable energy configuration [16], which results in magnetic navigation being able to be carried out at deeper locations inside the body [17].

There are multiple fabrication methods for synthesizing NWs. These include chemical methods, physical methods, electrodeposition, and electroless deposition [18], which use both the bottom-up and top-down approaches. In the top-down approach, which is a subtractive technique, material is carved of a larger starting material block, revealing the NW. On the other hand, the bottom-up approach is an additive-type synthesis in which smaller particles are bound together to synthesize the NW [19].

This review will focus on design of multifunctional systems of magnetic NW, including the fabrication methods of magnetic NW; strategies and application of magnetic NW-based nanosystems for cancer therapeutics; characterization of the magnetic NW nanosystems including toxicity, cell internalization, drug loading, and release; and critical evaluation of the performance for NW-based multifunctional nanosystems, for improved therapeutic outcomes.

## 2. Considerations and Applications of Magnetic Nanowires for Cancer Therapeutics

In order to design effective cancer therapeutic systems, the applications of the magnetic NW must be tailored to achieve in the appropriate microenvironment of the targeted cancer, which the therapeutic system is designed for. Thereafter, the most appropriate applications of magnetic NW must be synergistically combined to validate the rational of incorporating the NW into a multifunctional system. Below, the general considerations of tumor microenvironment will be discussed as well as the applications of magnetic NW in a therapeutic system.

### 2.1. Considerations of the Microenvironment of Cancerous Tissue for the Design of Magnetic Nanowire Therapeutic Systems

Tumor microenvironments play an important role in the biological impact of nanosystems as well their distribution [20]. For nanosystems to be efficacious for cancer therapeutics, it needs to attain a homogenous distribution intratumorally, however nanosystems need to overcome the tumor microenvironment’s barriers, which are summarized by Fernandez and co-workers [21]. Although the enhanced permeability and retention effect promotes extravasation of nanosystems intratumorally, they must first overcome the high interstitial pressure, abnormal tumor vasculature, and dense stroma, so that they may be efficacious. Explicit pathophysiological conditions of the targeted tumor, such as functional proteins and levels of amino acids, as well as endogenous factors of the tumor microenvironment must be considered in the design of optimal-nanosystems. These factors include acidosis, hypoxia, hyperthermia, oxidative stress, enzyme activity, redox potential, and high interstitial fluid pressure. However, these factors can also be exploited in the drug delivery design of nanosystems. For instance, nanosystems can be designed to take advantage of the tumor acidic environment, which differs from physiological pH to initiate drug release. This can be achieved by bonding the drug to the nanosystems using acid hydrolysis sensitive covalent bonds [22]. Active targeting can also be achieved using pathophysiological conditions of the targeted tumor. This is accomplished by binding specific antibodies that bind to receptors expressed on the tumor cell such as attaching anti-Her2/neu antibody to the nanosystem, which binds to Her2/neu receptors on the tumor cell membrane. Aptamers and ligands can also be used in a similar regard [23]. A promising potential application of magnetic NW drug delivery systems is the attachment of Wnt inhibitors. Traditionally, Wnt inhibitors are restricted by high toxicity and inefficient drug delivery systems [24]. However, these limitations can be overcome by a targeted and stimuli-release drug delivery system, which nanotechnology, and in particular magnetic NWs, can achieve [24].

### 2.2. Cancer Therapeutic Applications Employing Magnetic Nanowires

Nanocarriers have been greatly reviewed and have shown to have great therapeutic benefits. These advantages include the ability to increase the permeation of drugs across the epithelial lining of the gut wall, half-life, and solubility of hydrophobic drugs. Nanosystems, on the other hand, are favored due to its ability to overcome the limitations of conventional therapy. For example, being able to selectively release drug, increase accumulation in the target organ, and design a targeting ability within the nanosystems. Nanosystems also have the capability to perform multiple roles, such as theranostics, measuring dose response, and drug efficacy.

NWs can be integrated into such multifaceted drug delivery systems coalescing the inherent properties of NW and the efficacy of nanosystems, producing an advanced, modifiable, and functionalizable platform for drug delivery. These platforms are most commonly in a hybrid inorganic-polymer NW orientation or synthesized as a silicon core. The NW being the inorganic core while the surface coatings bring about a variety of biomedical properties. These surface coatings use various stimuli to illicit responses in a way that allows the systems to become targeted, selective, and stimulate drug release in order to increase therapeutic outcomes and decrease adverse effects of therapy.

This combination is effective in the development of drug delivery platforms for cancer treatment due to the unique merits it provides. It can enhance therapeutic effects by combating multiple drug resistance in cancers or provide a synergistic combination of therapeutic effects. This combination also allows for the accumulation of drug at the targeted tumor sites, thus reducing adverse effects of treatment [25,26,27,28].

#### 2.2.1. The Application of Magnetic Nanowires as Magnetic Drug Targeting Agents in Cancer Therapeutics

Drug accumulation at specific tumor sites can be achieved when an external magnetic field is used to draw out and trap magnetically active, drug-loaded nanoparticles from the circulatory system. It is promising for its potential of increasing the saturation of drug at the required site while decreasing the saturation in healthy tissue. Thus, reducing adverse effects and increasing therapeutic outcomes. Magnetic targeting is thus dependent on two factors, a nanocarrier that is magnetically responsive and a magnetic field gradient [29]. Magnet systems employed in magnetic targeting fall into two classes, the use of an external magnet and the combination of an external magnet with an implanted magnet near the target area [29]. Magnetic NW systems that are delivered into the blood stream must overcome the viscous drag force of the blood stream. Therefore, the magnetic NW systems will potentially be captured from the capillary blood flow by the external magnet to the target area. The large magnetic moments of magnetic NW reduce the field gradient required to capture the NW [17]. To provide the magnetic field and magnetic field gradient, there are currently two categories of magnet systems; static field magnet systems and varying field magnet systems. Static field magnet systems are low cost, convenient, and simple but lack targeting accuracy, while varying field magnet systems have high targeting accuracy, which make it possible for employing three dimensional (3-D) precise targeting but are energy consuming and require complex hardware systems and exact calculations [30].

Magnetic NWs have an inherent advantage over spherical nanoparticles such as superparamagnetic iron oxide nanoparticles, as the anisotropy of NW allow for deeper tumors to be targeted and have a higher drug loading capacity [12,31]. Pondman et al. created an iron-palladium (FePd) NW system functionalized with oleic acid. This resulted in non-immunotoxic, non-cytotoxic delivery platform granted, unsuccessful in accumulating the NW at the target site in pilot studies. Their FePd NW dimensions were 1.9 ± 0.3 µm in length and 88 ± 15 nm in diameter resulting in an aspect ratio of 22. When a magnetic field was applied to the NW inside the template in three different directions, 0°, 45°, and 90° to the NW direction, the wires showed remanence in all three directions and when tested in random orientations, provided a saturation magnetization (Ms) ± 80 A.m^2^/kg and a remnant magnetization (MR) ± 25 A.m^2^/kg [17]. Pondman and co-workers performed in vivo studies using Their FePd NW on rats. No negative reactions were shown after intravenous administration with no FePd NW found in the kidneys and liver. The studies suggested a high circulation time due to the immune response and first pass filtration of the kidneys not removing the FePd NW system. However, they were not able to prove significant localization of their NW system at target site. This was likely caused by the removal of blood from the rat in the fixation process [17]. Alsharif et al. iron (Fe) NW with an aspect ratio of 75 had a Fe_2_O_3_ layer surrounding the Fe NW and provided much larger Ms and MR of 427 A.m^2^/kg and 388 A.m^2^/kg, respectively. This confirmed its permanent magnetic properties and is indicative of its greater potential as a magnetic targeting agent when compared to the FePd NW. However, the Fe NWs will have a greater degree of aggregation, which will need to be overcome in order to be effective and safe as a drug delivery system [32]. The magnetic properties of the cobalt (Co) NW and functionalized Co NW of Zhu et al. was not characterized by a magnetometer. However, its potential for providing targeted chemotherapy was shown by suspending the Co NW, GO-Co NW, and GO-PEG-Co NW in polyvinyl alcohol (PVA) solution (where GO is graphene oxide and PEG is polyethylene glycol) and placed near an external magnet. This resulted in each group being attracted to the external magnet within one minute [33].

#### 2.2.2. The Application of Magnetic Nanowires as Hyperthermic Agents in Cancer Therapeutics

The use of NW as hyperthermic agents is promising due to its ability to be optimally structured to provide thermal response to stimuli such as low-frequency alternating magnetic fields and of near-infrared irradiation [34]. Hyperthermia involves the energy insertion into malignant tumors resulting in the death of the cancer cells. It can be characterized into three states; diathermy (greater than 41 °C), apoptosis (between 42 °C and 46 °C), and thermoablation (greater than 46 °C). Diathermy stimulates tumor growth, apoptosis is the ideal range for cancer cell destruction, while thermoablation stimulates heat-induced necrosis [35].

There are two modes of inducing magnetic hyperthermia in the presence of an alternating magnetic field. These are the Brownian relaxation mechanism and the Néel relaxation mechanism [36]. The Brownian mechanism involves the NW rotation-vibration towards the direction of the external magnetic field. This results in a mechanical friction caused by the magnetic NW in its suspended medium, inducing the hyperthermia. The Néel mechanism involves the rotation of the magnetic moment within the NW in an external magnetic field. Néel’s mechanism therefore induces hyperthermia by the “internal friction” caused by the magnetic moment movement. The Néel mechanism provides a more specific cell death mechanism as it induces minor mechanical damage to cells when compared to the Brownian mechanism, which is non-selective in its mechanical damage of cell membranes. In addition, heat induced in terms of hysteresis losses is dependent on the particular reversal mechanism. This is usually mediated by the nucleation and propagation of a magnetic domain wall. The domain wall is dependent on both the specific materials and geometry and can be of two types, vortex or transverse domain wall. The domain wall dynamics influences the heating performance of the NW and creates a certain maximum frequency in which a heating response is elicited [37]. Magnetic heating has strong dependence on the magnetic properties of the magnetic NW [38]. Specific absorption rate, which is used to quantify the heating efficiency, is increased when an alternating magnetic field equal to or lower than the coercive field is applied. Therefore, metallic NWs such as Nickel (Ni) and Fe have greater heating power when compared to Co due to their coersivity. The coercive field is also influenced by the geometry of the NW. Consequently, the heating efficiency of thicker NW will be greater than that of thinner NW and longer NW will be greater than shorter NW [38,39].

The recommended frequencies of electromagnetic fields lie between 50 kHz < *f* < 1 MHz as physiological responses such as muscle (skeletal and peripheral) and cardiac stimulation occur with increasing frequencies [40]. Choi et al. produced Ni NWs and successfully induced hyperthermia in HEK-293 cells. This was achieved using radio frequency (RF) electromagnetic fields. The Ni NW was internalized by the cells and after the application of a RF of 810 MHz [41]. Lin and coworkers fabricated Fe NW with a coercive force of about 9.7 Oe. This provided a high saturated heating temperature of 73.8 °C at a concentration of 500 ppm. During their cytotoxicity studies investigating hyperthermia derived from Fe NW, they revealed a mortality rate of 80% for EMT-6 cells. This highlights the feasibility of using Fe NW in hyperthermia therapy [36]. Alonso et al. synthesized FeCo NW to study their potential in magnetic hyperthermia. They found that the Specific absorption rate increased with an increase in length and obtained remarkable specific absorption rate values of ∼1500 W/g [39]. Hopkins et al. produced Ni-gold (Au) core-shell NW and for RF initiated hyperthermia for thermotherapy. During in vivo, the NiAu core-shell NW was intratumorally injected into the mice. A RF of 950 MHz and power of 10 W was then applied for 30 min with the mice under injectable anesthesia with a second and third treatment carried out at day 20 and day 30, respectively, after the first treatment. This resulted in significant damage to the malignant solid tumor on the mice [42].

#### 2.2.3. The Application of Magnetic Nanowires as Magnetic Actuation Agents in Cancer Therapeutics

**Figure 2:**
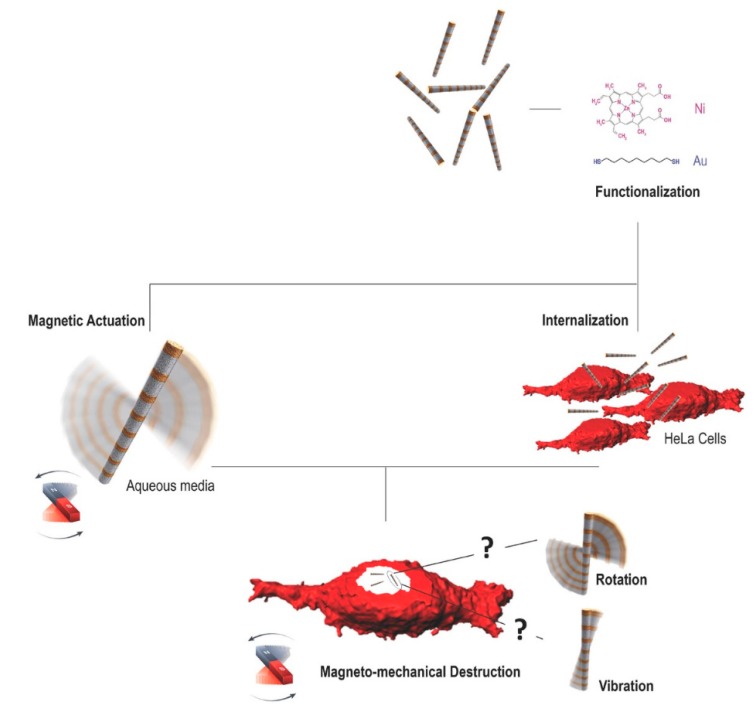
Diagram showing proposed mechanism of action for magnetic actuation stimulating a magneto-mechanical cell death in the presence of an alternating magnetic field. Adapted with permission from [46].

Magnetic NW can induce cell death without a heat dependent mechanism in a magneto-mechanical process as depicted in Figure 2 [43,44]. The first study of magnetic actuation induced cytotoxic effects arising from alternating magnetic fields at low frequencies was studied by Zablotskii and colleagues [45]. They applied a high-gradient magnetic field with a low frequency (1–10 Hz) as well as mechanical vibration on incubated mesenchymal stem cells. Their results suggested that both the mechanical vibration and alternating magnetic field played an active role in the F-actin remodeling and succeeding down-regulation of the audiogenic genes adiponectin AP2 and PPARγ.

This mechanism was later applied to a more cancer therapeutic approach by researchers. The exemplary study of Contreras and co-workers exhibited the use of Ni NW for a non-chemotoxic approach to cancer cell death. They fabricated Ni NWs with a length 4.1 ± 1.4 µm and a diameter of 30 to 40 nm. The Ms value measured was 46.7 A.m^2^/kg, which is lower than the reported literature value for bulk Ni, which is 54.3 A.m^2^/kg [47]. This phenomenon was associated with the surface oxidation of the Ni NW according to Contreras and co-workers. When comparing the array Ni NW to a single Ni NW, the Ms increased to 47.4 A.m^2^/kg as the single Ni NW acts as a permanent magnet and is free from magnetostatic interactions, which the array experiences and thus show single domain properties [48]. The behavior of magnetic NW is administrated by its magnetization in the presence of an alternating magnetic field. In the case of Ni NW, it is determined by the shape anisotropy and the NW axis (magnetic easy axis) [44,49]. This results in the Ni NW to produce a torque when trying to align their magnetic moment with the alternating magnetic field. This mechanism is applicable for all magnetic NW with the same characteristic. Therefore, when the NWs are exposed to an alternating magnetic field, they will experience torque, while trying to align the magnetic moment with the field. This torque results in a force being applied on the cell, which leads to its death in the presence of an alternating magnetic field as shown in Figure 2. Serrà et al. fabricated a multi-component Au/Ni–nickel oxide (NiO) NW using pulsed potentiostatic electrodeposition. They incubated the NW with HeLa cells for 24 h, after which 70% of the NW were internalized. They observed 24% cell death after an alternating magnetic field of 14 and 35 mT and 20 Hz was applied for 15 min. The segmentation of the NW assisted in tailoring the MR, which in turn decreased the NW agglomeration, and the observed cell death was not induced by magneto-mechanical effect due to the lower MR, but rather it was associated with the NW vibration, which further highlights the association of magnetization and NW behavior [46].

Specific loss power (heat produced) frequency dependence is linear for ferromagnetic particles [50]. Therefore, in order to produce the heat required for thermoablation, the amplitude of the alternating magnetic field necessary is ~10 kA/m and around 100 kHz frequency is required [51,52]. Magnetic actuation cell deaths were induced at ranges largely below those thresholds. The Fe NW systems of Martínez-Banderas and co-workers used a 1 mT, 10 Hz alternating magnetic field to induce cell death while Contreras and co-workers used alternating magnetic fields of 0.5 mT and 0.1, 1, and 10 Hz to induce cell death. This low amplitude and frequency requirement translate into lower cost of magnetic actuation cancer treatment and increased safety of patients by reducing the risk of thermoablation [43].

This principle of inducing magneto-mechanical cell death by magnetic actuation can be applied to cancer therapeutics to induce non-chemotoxic destruction to a malignant tumor. The advantage of this is that it not only can reduce or eliminate chemotherapeutic side effects, it can also be used as an alternative cell killing mechanism in multi-drug resistant cancer.

### 2.3. Magnetic Multifunctional Nanowire Systems in Cancer Therapeutics

The core principle behind formulating NW systems using a magnetic core is magnetic navigation [17,53,54]. The ability to localize a delivery system using a non-invasive and relatively safe force is highly remunerative in cancer therapy, as it allows the progression from the limitations of traditional cancer chemotherapy by allowing therapeutic effects to be directly targeted at the tumor site, thus reducing secondary effects. In addition to magnetic navigation, magnetic wires are used for inducing cell death by magneto-mechanical means using magnetic actuation [43] and induced local hyperthermia [55] by applying a low- and high-frequency alternating magnetic field, respectively.

**Figure 3:**
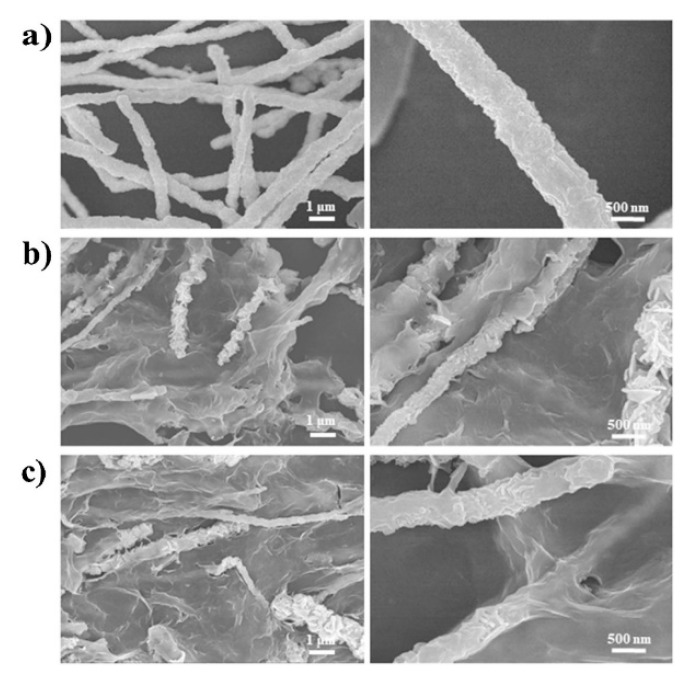
(**a**) Microscopy of unfunctionalized Co nanowires (NWs) portraying a rough morphology. (**b**) Microscopy of stimuli responsive graphene oxide (GO) functionalized Co NW. (**c**) Microscopy of GO-polyethylene glycol (PEG)-functionalized Co NW. Reproduced with permission from [33].

Magnetic NW cores are functionalized by their surface modifications. These modifications alter the pharmacokinetics of the magnetic NW systems, adjust the cytotoxicity, allow for attachments of biomolecules such as ligands, and allow for the conjugation or entrapment of drugs [56]. Surface modifications are also used to input a responsive behavior to the system usually to provide effects such as triggered drug release or hyperthermia. The stimuli used to initiate such behavior include a change in pH and radiation. The coalescence of the magnetic NW and stimuli-responsive surface coatings create multifunctional magnetic NW systems that have potential advantages over traditional cancer therapy. Figure 3a depicts Co NWs while Figure 3b,c depict Co NWs with surface modifications that were designed to increase the biocompatibility, enable drug loading using electrostatic means, and provide photothermal responsiveness from a stimulus (near-infrared irradiation).

#### 2.3.1. The Use of Magnetic Nanowire Magnetic-Chemo-Photothermal Systems in Cancer Therapeutics

The use of a multifunctional system that is magnetically targeted to deliver a chemotherapeutic load to the tumor site, in addition to inducing a local hyperthermia in the tumor to induce cell death in synergy to the chemotherapeutic drug, is advantageous for the following reasons; it decreases drug adverse and side effects, enhances the efficacy of tumor destruction, and can potentially be used to combat multi-drug resistance in chemotherapy. This approach was explored by Zhu et al. [33]. They built this multifunctional nanowire system on Co nanowires treated with GO and PEG, which provided the desired responsiveness to pH, magnetic fields, and near-infrared irradiation. A template-free reduction method was used to synthesize the CO NWs in the presence of a magnetic field. This produced unordered, irregular, and rough NWs on which the PEG and GO were coated. Three different groups were produced, namely Co NWs, Co NWs-GO, and Co NWs-GO-PEG, and were characterized by their drug loading ability, toxicity magnetic, and photothermal properties, which are discussed in the relevant sections below. They produced a system that can potentially be magnetically targeted to a tumor site, thereafter release the loaded drug due to the combined decrease in PH at the tumor site, and an application of near-infrared irradiation, which both stimulates drug release and local hyperthermia; thus, inducing cell death by both the drug and the local temperature increase. This is a promising model; however, further studies need to be carried out to determine the in vitro and in vivo magnetic targeting ability so that this system can be phased into clinical trials.

#### 2.3.2. The Use of Magnetic Nanowire Magnetic Actuation-Chemotherapeutic Systems in Cancer Therapeutics

Martínez-Banderas et al. conducted a study, which used the combined chemotherapeutic effects of doxorubicin (DOX) and mechanical disturbances, induced by a low-frequency alternating magnetic field, of their magnetic NWs to prompt cancer cell death. Their system was built upon Fe NWs with an average length of 6.4 ± 1.3 μm and a diameter of 30 to 40 nm. The Fe NW were coated with bovine serum albumin (BSA) and (3-aminopropyl) triethoxysilane (APTES) independently and additionally functionalized with DOX. The Fe NW were synthesized using electrodeposition and formed NWs of a monocrystaline nature. An interesting aspect of these Fe NWs was that it was found to have a 4–10 nm thick layer of iron oxide on its surface (monocrystaline Fe NW surface oxide layer caps at 10 nm) [57] as compared to completely oxidized, which occur to polycrystalline Fe NW over time. This ensures that an Fe core will remain. The oxidation most likely occurred when dissolving the template in sodium hydroxide (NaOH) as the NaOH provides very good oxidizing conditions for Fe. The Fe oxide interphase is important for two reasons. The first being that the remnant magnetizations and magnetic saturation depend on the oxide thickness. The second being it provides a site for the covalent attachment of surface coating. Martínez-Banderas and colleagues thus created a system in which cell death was caused by the cytotoxic effect of DOX and structural damage to the cells by the magneto-mechanical disturbances as shown when tested on MDA-MB-231 cells [11,58].

#### 2.3.3. The Use of Magnetic Nanowire as a Theranostics System in Cancer

It is important to note the role of magnetic NW in theranostics, a paradigm shift promoting personalization of treatment through the combination of diagnostics and therapeutics. The role of magnetic NW platforms in therapeutics encompasses targeted and triggered release drug delivery, photothermal therapy, and magnetic actuation systems as discussed above [59]. In terms of diagnostics, the large surface area of NW increases the efficacy of fluorescence labelling while in terms of magnetic NW, it increases the magnetic moment, making it attractive in fluorescence imaging and magnetic resonance imaging (MRI), respectively. Contrast agents for MRI are grouped into two categories, namely T_1_ and T_2_. The distinguishing factor between T_1_ and T_2_ agents is that T_1_ is based on longitudinal magnetization recovery while T_2_ is based on transverse magnetization decay [60]. In this regard, Fe and Ni NW have been shown to be useful T_2_ contrast agents with Ni NW being comparable to commercial agents [61,62]. The effectiveness of NW in both therapeutics and diagnostics makes NW and magnetic NW an attractive prospect for designing theranostics systems for cancer.

## 3. NW Fabrication and Synthesis of Magnetic Nanowire Drug Delivery Systems

### 3.1. Fabrication Methods of Magnetic Nanowires

There are two main approach strategies for the fabrication of NW. Namely, the bottom-up and top-down approaches. The bottom-up approach involves the spontaneous assembly of small substrates (atoms or molecules) into the desired nanostructure while the top-down approach involves the breakdown of a suitable starting material until the desired nanostructure is formed [63].

A common top-down technique is lithography. Lithography is further divided into various techniques including photolithography and electron beam lithography. The main principle of these techniques is that a pattern is engraved onto an underlying substrate and the desired material is transferred onto the pattern. Therefore, these techniques are a hybrid of the top-down and bottom-up approaches.

These techniques have the advantage of easily being scaled up and can be used for the production of one- and two-dimensional particles that has at least one lateral dimension in the nanoscale range, however the resolution achieved is low and has etching and coating constraints, thus making it impractical for the use in drug delivery [63,64].

Bottom-up techniques enable the synthesis of complex structures such as non-straight vertical structures, structures made up of multiple components, and structures that have changes in the chemical composition. The main advantage provided by bottom-up techniques is the ability to synthesize structures with high aspect ratios. Bottom-up techniques employ chemical [33,65], physical [66], and electrochemical [67] methods to produce nanowires.

The NW fabrication techniques are summarized in Table 1, noting the advantages and disadvantages of each while selected fabrication techniques of promising methods used for the NW synthesis in drug delivery are discussed in further detail below, further highlighting the advantages and disadvantages of the technique and evaluating the potential for each technique to advance drug delivery.

#### 3.1.1. Electrodeposition of Magnetic Nanowires

A very convenient and common method for nanoparticle synthesis is electrodeposition. It was traditionally used as a conventional surface modification method for adjustments of surface morphology and characteristics and can be used to fabricate nanoparticles of single or multiple composition [78,79,80,81]. Electrodeposition is the formation and deposition of solids through electrochemical reactions. These solids are usually formed by the reduction of an electroactive species contained in an electrolyte by applying a potential. This distinguishes it from electroless deposition in which a reducing agent replaces the applied potential [82].

A further distinction can be made in the electrodeposition of NW; deposition using templates and template-less deposition [70]. There are two types of templates, soft templates and hard templates. Soft templates are non-rigid structures that are used to control the direction of growth and therefore resultant shapes of nanoparticles produced. Soft templates include surfactant aggregates, micelles, and co-block polymers, and can be used to generate porosity and texture control of the resultant NW. The downside of soft templates is that the baths containing the soft templates usually express a low conductivity, which in turn hinders the electrodeposition process. Hard templates are rigid structures that are conductive on one end and regulate the size and shape of the synthesized nanoparticles by the shape of the template itself. The pioneering work of Possin first demonstrated this principle of fabricating small diameter NW in porous membranes [83]. Hard templates are extremely versatile, convenient, and are recurrently used in the synthesis of NW. The most common hard templates are anodized alumina, polycarbonate membranes, and mesoporous materials [84]. Hard templates allow for the synthesis of freestanding NW as well as both orientated and non-orientated NW. They also facilitate the synthesis of complex one-dimensional NW [70]. The downside of using hard templates include the removal of the template as it can affect the NW, diffusion of the electroactive species through the narrow diameter pore channels, difficulty in upscaling due to the difficulty of producing large templates with homogenous pore distribution (with small pore diameters), and the difficulty of producing templates with uniform pore diameters. When no physical template is used for controlling the morphology and shape of the produced nanoparticle, deposition rates are used in its place. Control of deposition rates is achieved by the modification of current density concentration of the electroactive species, temperature, and applied potentials.

Electrodeposition therefore has the potential of producing NW for effective drug delivery systems as precise control of the NW dimensions can be achieved, which is essential for positive cell interaction, control of magnetic ability, and drug loading. This technique can produce a variety of non-toxic, biocompatible NW including iron oxide, iron, and iron–palladium NWs, as well as segmented NWs in a convenient, scalable, and reproducible way; thus, providing a stable platform in which drug delivery systems can be engineered onto while keeping the inherent benefits of a NW for multifunctional systems.

#### 3.1.2. Pulsed Laser Deposition of Magnetic Nanowires

Another common and valued growth mechanism for the synthesis of nanowires is pulsed laser deposition (PLD) [85,86,87,88]. A pulsated high-powered laser beam is used to excite the surface energies of a target substrate in a controlled atmosphere producing an ejected plume. The vapor is then deposited onto a sample stage producing thin films or nanoparticles with the same composition as the target. PLD allows for synthesis of nanoparticles with a monocrystalline nature, high purity, and low defects to be synthesized. Other advantages of PLD is that it has a fast production rate and it is scalable.

Shkurmanov and co-workers studied the growth of zinc oxide (ZnO) nanowires via PLD in order to understand the mechanism in which the nanowires are formed [89]. They observed that the NW growth was non-linear and can be explained in terms of the number of laser pulses applied and its interaction with four distinct flows of particles. The first flow forms the nuclei in which the NW will grow from, while the second flow is responsible for the vertical growth of the nanowire. The third flow was found to cause a backflow and decreased the length of the NW. Lastly, the fourth flow was responsible for the lateral growth of the NW. This proved that geometric parameters can be controlled when using PLD.

An exemplary study of Nikov et al. paved the way for the formation of magnetic NW using PLD by employing the aid of a magnetic field [87]. This study provided a simple method of producing magnetic NWs, which increases its industrial value. This method also produces NWs composed of smaller nanoparticles. This property can be exploited to potentially positively affect drug loading capabilities of drug delivery systems. Furthermore, Nikov and co-workers used this method to fabricated iron oxide NW [90]. This study further demonstrated that the pressure and surrounding gas can be used to manipulate the type of oxide formed. Another interesting effect of the magnetic field is that it allowed the deposition of the iron oxides in arranged nanowire structures at a low pressure and a target substrate distance of 40 mm.

PLD provides an effective method of producing NW arrays, which are especially promising in implantable and transdermal drug delivery systems. It excels in producing NW composed of magnetic materials, which is a property that can be exploited in the development and commercialization of multifunctional drug delivery systems.

#### 3.1.3. Other Synthesis Techniques of Magnetic Nanowires

There are various other fabrication techniques in which NWs can be synthesized. However, the most promising fabrication technique is electrodeposition due to its precise dimension control. These techniques are commented on in Table 1 and include atomic layer deposition, chemical vapor deposition, pulsed laser deposition, focused electron beam induced deposition, chemical reduction, solvothermal, hydrothermal, sol-gel, and lithography techniques such as photolithography and electron beam lithography.

### 3.2. Magnetic Properties and Advantages of Nanowires in Drug Delivery Systems

NW are synthesized from both magnetic and non-magnetic substrates. Both NW and magnetic NW offer potential advantages over nanoparticles and magnetic nanoparticles due to their larger surface area to volume ratio (high aspect ratio), which allows for greater drug loading, increased attachment sites, for decorations such as proteins, peptides, and polymers, and increased binding to cells. Magnetic NW have greater advantages when compared to magnetic nanoparticles due to their strong shape anisotropy and energetically favorable magnetization. They provide greater magnetic moments and in the presence of an alternating magnetic field, can either provide mechanical motion by aligning to the magnetic moment with an applied low-frequency alternating magnetic field or induce a local hyperthermia at a high-frequency (~100 kHz) alternating magnetic field [11].

The most frequently used materials for synthesizing the magnetic components of magnetic NWs for drug delivery systems are Fe, Ni, Co, as well as their compounds and alloys. Magnetic NWs display specific advantages when compared to spiracle and other nanoparticles as well. The increased aspect ratio of ferromagnetic NWs provides stronger magnetic moments per unit volume and large remnant magnetizations without decreasing the mobility of the nanoparticles. The larger remnant magnetization allows the NW to be used in low-field environments, which in turn translates into NWs being able to target deeper tissues with smaller and weaker magnets as the geometry of NWs has an increasing effect on force applied by the magnetic field. NWs with aspect ratios greater than three show larger magnetic dipoles when compared same volume spherical nanoparticles [12]. This results in the potential of a more efficient magnetic system to be designed for magnetic drug targeting for cancer therapeutics.

### 3.3. Stabilization and Functionalization of the Magnetic Nanowires

The surface area of NWs are decorated with coatings mainly for three intended purposes: To increase the biocompatibility of the NW, stabilize the NW (prevent NW agglomeration), and to functionalize the NW in order to tailor the NW to excel in the niche of interest [43].

The work by Zhu et al. used a coating of PEG and GO to both stabilize and functionalize their Co NW, thereby increasing its drug loading capacity and biocompatibility. Both the PEG and GO were attached to the Co NW by electrostatic adsorption using an ultrasonic dispersion method. The GO played a dual role; to enhance the photothermal therapy efficacy, as it is a known photothermal agent, and to provide attachment points for the loaded drug (Doxorubicin) [33]. When irradiated with a 808 nm laser for six minutes, the Co NW and GO-functionalized CO NW heated to a temperature of 39.1 °C and 40.6 °C, respectively, indicating the ability of GO to improve the excellent photothermal effect of Co NW.

Magnetic NWs can also be functionalized by the attachment of antibodies to target specific cells, thus increasing its selectivity. Contreras et al. successfully functionalized their Ni NWs with EGFR antibody (ab62 abcam^®^). This was achieved by first modifying the antibody with N-Succinimidyl S-acetyl thioacetate. Thereafter, it was further modified to introduce sulfhydryl groups so that it could attach to the Ni NW [91].

**Figure 4:**
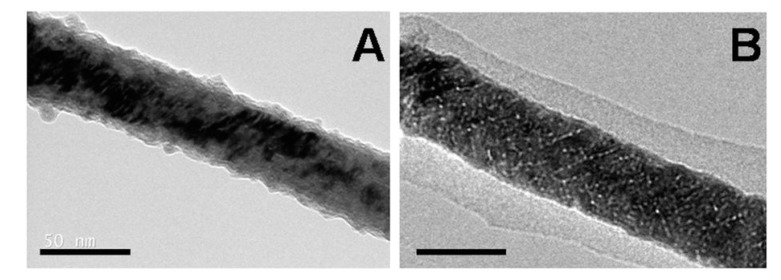
Figure showing surface modifications on magnetic NW for stabilization. (**A**) (3-aminopropyl) triethoxysilane (APTES)-coated Fe NW. (**B**) Bovine serum albumin (BSA)-coated Fe NW. Reproduced with permission from [11]. Scale bars: 50 nm.

The NW system of Martínez-Banderas and research group was tested with three distinct coatings, BSA, APTES, and APTES-PEG. The BSA and APTES were covalently bonded to the surface Fe_2_O_3_ interphase of the Fe NW while they functionalized APTES with PEG by activating the APTES with sulfhydryl groups and the reacting with the thiol group in thiol-PEG, thus achieving disulfide bonds. The three coatings were compared using MDA-MB-231 breast cancer cells, which were incubated with the coated Fe NW and added cyanide Fe salt. Bright field imaging was used to determine the distribution, size, and morphology of the Fe NW agglomerates. All three coatings reduced the size of the agglomerates, thus ensuring a greater homogeneity of the Fe NW distribution across the sample. APTES-PEG had the least efficacy of this effect [11]. Figure 4A,B depicts the transmission electron microscopy image of the APTES-NW and BSA-NW respectively, visualizing the coating on the NW.

Contreras and co-workers did not stabilize their Ni NWs with any coating, which thus caused the aggregation of the Ni NW. The Ni NW zeta potential was measured to be a low value of −15.1 mV [92]. This value infers a weak electrostatic repulsive force which correlates with Contreras’ observation of released Ni NW aggregating. This study highlights the efficacy of surface coatings to stabilize NW in terms of aggregation of the NW. Preventing the NW tendency to aggregate is essential as particle aggregates in the bloodstream can cause an embolism, while in tissue it can cause heterogeneous cytotoxic activity. Martínez-Banderas and research group managed to reduce the aggregation of Fe NWs, which is known to have higher remnant magnetization, which makes it inherently unstable. The stability of the Ni NW can be increased with surface modification such as coating with charged polymers or non-magnetic metal such as Au. The increase in magnetic NW stability increases its desirability in drug delivery as it will allow for safer therapy a formulation with a longer shelf life.

Stabilizing NW is therefore indispensable in the design and formulation of NW drug delivery systems as it prevents the aggregation of magnetic cored NW caused by their remnant magnetization. This, in turn, reduces the probability of mechanical obstruction in the circulatory system caused by NW aggregation. Therefore, NW coatings allow for the optimal design of multifunctional systems, which is essential in potentially improving cancer therapeutic outcomes.

### 3.4. Chemotherapeutic Drug Loading and Release of Magnetic Nanowire Systems

Large surface area has a positive influence on the drug loading capacity of nanosystems. Thus, NW morphology has a direct impact on drug loading capacity. Although NWs inherently have large surface areas, their surface area can be increased further by changing their morphology to include rough surfaces [93,94] or by synthesizing porous NWs [95,96]. Guo and co-workers achieved a high drug loading of 2000 mg/g using porous NW while Zhu and co-workers achieved a high drug loading capacity of 992.91 mg/g with their Co NW, which had a rough morphology. The GO in the functionalized Co NW of Zhu and research group provided attachment points for DOX or other therapeutic agents as it is decorated with many functional groups on its surface such as hydroxide radicals. In the case of DOX, it is hypothesized that it is also able to absorb directly onto the GO via π-π interactions. Their NW system also exhibited a higher drug release profile in acidic environments and after near infrared radiation when compared to the control [33]. The decrease in PH made the DOX more hydrophilic and soluble due to the protonation of the NH_2_ group on it, thus causing the release from the Co NW-GO. They also proved a direct correlation between drug release and laser power intensity.

The BSA and APTES coated Fe NW of Martínez-Banderas and colleagues were functionalized using PH responsive covalent bonds. This was achieved by introducing free thiol groups to the coated Fe NW by reacting 2-IT and amine groups on the coated Fe NW. The free thiol groups were then reacted to the maleimide group of a DOX derivative (5-Maleimidovaleroyl) hydrazone of Doxorubicin in order to attach it. This yielded low loading capacities of 50 μmol DOX/g Fe (27 mg/g) for the DOX-APTES-Fe NW and 25 μmol DOX/g Fe (13.6 mg/g) in the case of DOX-BSA-Fe NW [11]. The low loading was due to the relatively smooth surface of the Fe NW, highlighting the importance of morphology on drug loading.

## 4. Cellular Interactions and Toxicity Between Magnetic Nanowires and Cells

### 4.1. Cellular Internalization of Magnetic Nanowires

Cellular internalization of NW supports the NW utilization and efficacy. There are three main potential uptake mechanisms for nanoparticles; receptor-mediated endocytosis, pinocytosis, and phagocytosis [97]. NW suffers an inherent disadvantage when compared to spherical nanoparticles in terms of cell internalization. It is speculated that the different curvatures between the two shapes has a direct effect on cell binding. When the longitudinal axis of NW is bound to the cell membrane, the larger surface contact area blocks available membrane receptors, thus reducing cell internalization. There are many factors that govern cell internalization of nanoparticles besides shape. These are extensively discussed in a review conducted by Murugan et al. of the Wits Advanced Drug Delivery Platform, South Africa [98]. Briefly, in order to design smart nanosystems, an understanding of physicochemical properties is essential. Other determinant factors in terms of cell internalization include uptake pathways and interaction of the nanoparticles with receptors. Neutral and cationic nanoparticles have higher transport efficiency into the cells when compared to negatively charged nanoparticles [99,100]. This phenomenon is the result of anionic particles having smaller binding efficiency to cell surfaces when compared to cationic and neutral particles, leading to a reduction in membrane-wrapping phenomena resulting in a decrease in cellular internalization [99,101,102]. Hydrophilic outer protective layers increase circulation time while nanoparticles functionalized with proteins or peptides directly increase cellular uptake by localizing the nanoparticle at the targeted site, receptor-mediated endocytic pathways, and direct cell penetration. Nanoparticle size also plays an important role in cellular uptake; 95–200 nm is the ideal size according to the literature for increased cellular uptake [103]. In terms of NWs, high aspect ratio NWs can also be internalized [104].

**Figure 5:**
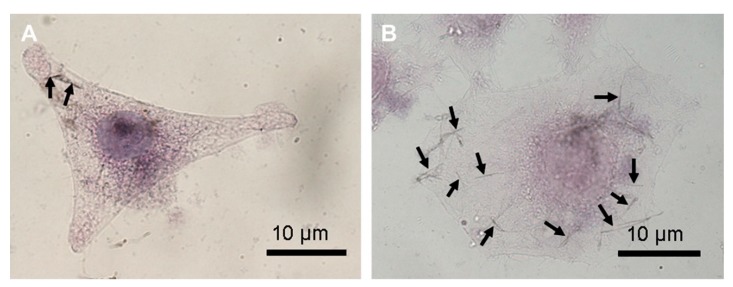
Cellular internalization and accumulation of magnetic NW into cells. (**A**,**B**) Internalized Fe NW in HeLa cells. Adapted with permission from [105]. Scales bars: 10 µm.

Fe NW also have good cell internalization as shown by [105] and further demonstrated by Martínez-Banderas and co-workers. Martínez-Banderas and co-workers had a total cell internalization for their APTES-Fe NW and BSA-Fe NW of 19% and 15%, respectively. These values were determined using Inductively Coupled Plasma Mass Spectrometry (ICP-MS) measurements on their incubated cells with the coated Fe NWs. The results yielded no significant difference for the APTES-Fe NW and BSA-Fe NWs in the cellular internalization [11]. Figure 5A,B shows the cellular uptake using HeLa cells of uncoated, positively charged, non-uniformly sized Fe NWs of Song and co-workers. The weakly negatively charged Ni NW of Contreras and research group also had a high affinity to cell internalization. Similar results were achieved for other Ni NWs, including those with large lengths [106]. The cellular uptake of FePd NW of Guo and co-workers was studied on RAW264.7 and HeLa cells. Both cell lines took up both single NW and NW clusters with RAW264.7 cells having greater cell internalization, suggesting rapid removal from blood stream.

### 4.2. Cellular Toxicity of Magnetic Nanowires and Drug Loaded Magnetic Nanowires

Toxicity is important when designing nanosystems. The coatings used in NW systems are usually well-known biocompatible molecules used to counter the toxicity of toxic inorganic NW. Common NWs used in NW systems are silicon, Au, silver, Co, Fe, and Ni, amongst which Co, Fe, and Ni and their compounds produce magnetic NWs. From these, Co and Ni are the most toxic while Fe and silicon are deemed biocompatible. However, in comparison to Fe NW, a large number of studies have been conducted on Ni NW despite Ni’s known cytotoxicity, carcinogenicity, and genotoxicity [107,108], and the literature reveals that Fe is less toxic. The reason for this is that the large remnant magnetization of Fe NWs causes stronger aggregation and thus limits its use in the single NW form. Metal alloys are also used to synthesize NWs, such as iron palladium (FePd) NWs. FePd NWs are non-toxic and do not readily undergo oxidation [17,109]. Si NWs are also known to be non-toxic, have high biocompatibility, improve hydrophilicity, and can be made magnetic by decorating with Fe oxide nanoparticles. In terms of the effect of the NW length on cellular toxicity, Donaldson et al. reviewed the effect of fiber like particles and found that longer fibers were more toxic then shorter fibers [110]. However, Song et al. showed that at the same concentration, Fe NWs of shorter lengths (2 μm) were more toxic then longer lengths (5 μm) of Fe NWs [105]. According to Song et al., this was attributed to smaller size particles having greater cellular internalization [111].

The toxicity of magnetic NW can be altered by surface modifications of the NW. Zhu et al. conducted biocompatibility tests of their GO and PEG decorated Co NW by determining its cytotoxicity using 3T3 and 4T1 cells and hemocompatibility by determining if the Co NWs, Co NWs-GO, and Co NWs-GO-PEG caused thrombosis or hemolysis. The Co NW itself was found to be very toxic. However, the addition of GO and PEG decreased the cytotoxicity of the system to a great extent. The cell viability of 3T3 and 4T1 cells after culturing with the NW was 35% and 32%, respectively, for the Co NW, while it increased to 96% and 89%, respectively, for the Co NWs-GO-PEG for both at 50 μg/mL concentration. Their results also show no significant difference in the thrombosis time and did not cause hemolysis [33].

Applying an alternating magnetic field to magnetic NW while it is incubated with cells increases the toxicity of the NW. When incubating their Ni NW with HCT116 cells, Contreras and co-workers measured no significant drop in cell viability using 2.4 μg/mL of Ni NW without the presence of an alternating magnetic field, while at a concentration of 12 μg/mL, the cell viability dropped to slightly below 90%. When an alternating magnetic field is applied at 1 Hz, 0.5 mT for 1 h, the cell viability for 2.4 μg/mL drops to around 75% while using a concentration of 12 μg/mL causes the cell viability to drop to slightly below 70%. This shows that higher concentrations of magnetic NWs have a greater effect at inducing cell death [43].

The cellular toxicity of magnetic NWs increases when functionalized with cytotoxic agents both with and without the presence of an alternating magnetic field. Martínez-Banderas and co-workers conducted cell studies for their Fe NW systems using Alamar Blue assay and MDA-MB-231 cells to determine their systems ability to induce cell death in cancer by combining the cytotoxicity of DOX and the mechanical motion provided by the low-frequency alternating magnetic field. Their results showed that alternating magnetic field without the Fe NW systems had no effect on the cell viability and cytotoxicity of DOX. The coated Fe NW systems without the alternating magnetic field present had no significant decrease in the cell viability, which is testament to its biocompatibility while when in the presence of an alternating magnetic field produced a significant decrease in cell viability of 23% and 28% for the and APTES- Fe NW (26 μg of Fe/mL) and BSA-Fe NW (28 μg of Fe/mL), respectively, showing possible cancer cell death by Magnetic actuation. For the DOX-Fe NW systems, they showed a decrease in cell viability of 54% and 58% for DOX-APTES-Fe NW (26 μg of Fe/mL, 1.3 μM DOX) and DOX-BSA-Fe NW (28 μg of Fe/mL, 0.73 μM DOX), which is indicative of their selective intracellular drug release and the efficacy of Fe NW as nanocarriers. The addition of an alternating magnetic field to the DOX loaded Fe NW systems yielded a further 10% and 8% reduction in cell viability for DOX-APTES-Fe NW (26 μg of Fe/mL, 1.3 μM DOX) and DOX-BSA-Fe NW (28 μg of Fe/mL, 0.73 μM DOX), respectively, showing a weak additive effect instead of a synergistic effect for the combination of chemotherapy and magneto-mechanically induced cancer cell death [11].

It is interesting to note that although the BSA-Fe NWs had a lower drug loading capacity compared to APTES-Fe NWs, their efficacy in cytotoxic activity was similar. This was attributed, according to Martínez-Banderas and research group, to the greater cell internalization of the BSA-Fe NWs. Another point of interest is that NW can be used for the separation of biomolecules, their purification, and manipulation, which is applicable in the development of biosensors. This technology can lead to the ability to effectively detect circulating tumor cells leading to a better prognosis for cancer patients [112].

### 4.3. Cellular Degradation of Magnetic Nanowires

Understanding carrier degradation or metabolism is important in nanoparticle drug delivery to ensure high therapeutic efficacy [113]. The innovative work by Safi et al. revealed that cells are able to degrade NWs as well as decrease the size of the aggregates with their remains found directly dispersed in the cytosol or in vesicular compartments. Singular NWs, on the other hand, were found in endosomal compartments only [114]. Fe NWs and Ni NWs have oxidized surfaces and continue to be oxidized, degraded, and dissolved intracellularly by the lysosomal compartments. Perez et al. reported that ~2% of the Ni NWs dose was dissolved intracellularly after 71 h [115] and Fe experienced the same fate with the lysosomes without cytotoxic contribution. The fate of both the degraded and non-degraded NWs is potentially renal clearance. High aspect ratio nanoparticles have been found to be renally clearable with high efficiency of elimination. This is caused by the flow orientation of high aspect ratio nanoparticles, which aligns the long axis of the nanoparticle to point to the glomerular capillary pore openings [116].

## 5. Conclusions and Future Prospects

The application of magnetic NW has great potential for improving therapeutic outcomes for cancer therapy. They provide the benefits of traditional nanoparticles in addition to the inherent advantages of NW, thus making NW an ideal platform to build multifunctional nanosystems upon. However, the full potential of magnetic NW in drug has yet to be reached as further applications and functionalizations that can be molded onto the versatile magnetic NW platform such as Wnt inhibitor delivery systems, attachment of ligands in order to increase the selectivity, and a theranostic system that includes the attachment of fluorescent compounds, chemotherapeutic drugs, and modifications that increase cancer cell selectivity, require further research. Effective in vivo studies for multifunctional NW nanosystems is essential for the progression of this technology into the pre-clinical phase. The magnetic applications of NWs such as magnetic hyperthermia, magnetic actuation, and magnetic drug targeting has shown great potential for improving cancer therapeutics and therefore requires further research to optimize an efficacious multifunctional therapeutic system that will better the prognosis and increase the quality of life for cancer patients. Improvements can be made in tumor targeting so that magnetic NW can be accumulated into the tumor using a three-dimensional targeting model. The control of temperature change needs to be studied and optimized in in vivo studies.

## Figures and Tables

**Table 1:** Overview of fabrication techniques applied for the synthesis of magnetic NWs.

Fabrication Technique	Top-Down/Bottom-Up	Magnetic NW Composition Achievable by This Technique	Remarks on Technique	References
electrodeposition	bottom-up	Fe and Fe-based compounds Co and Co-based compounds Ni and Ni-based compounds	Cost effective enables synthesis of accurate dimensions and complex structure Individualistic growth on non-planar surfaces	[68,69,70]
atomic Layer deposition	bottom-up	Fe and Fe oxides Co and Co oxides Ni and Ni oxides	Cost effective enables complex structure synthesis Slow deposition rate	[70,71]
chemical vapor deposition	bottom-up	single-crystalline Ni and Ni alloys single-crystalline Co and Co alloys single-crystalline Fe and Fe alloys	Cost effective enables complex structure synthesis Difficult to deposit multicomponent constituents	[70,72]
pulsed laser deposition	bottom-up	Fe and Fe oxides Co and Co oxides Ni and Ni oxides	Cost effective enables complex structure synthesis Difficult to control dimensions of NW	[70,73]
Focused Electron Beam Induced Deposition	bottom-up	Fe deposits Co deposits	Cost effective enables complex structure synthesis Lack of control in final composition of NW	[70,74]
Chemical reduction	bottom-up	Fe and Fe-based materials Ni and Ni-based compounds	Cost effective Difficult to control NW dimensions and morphology	[70,75]
Solvothermal	bottom-up	Co and Co-based compounds Ni and Ni-based compounds Fe and Fe-based compounds	Difficult to control NW dimensions and morphology Requires high temperatures and pressures	[70,75]
Hydrothermal	bottom-up	Fe and Fe-based compounds Co and Co-based compounds Ni and Ni-based compounds	One step synthesis Difficult to control NW dimensions and morphology	[70,75]
Sol-Gel	bottom-up	Fe and Fe-based compounds Co and Co-based compounds Ni and Ni-based compounds	Cost effective scalable Can form defects in products	[70,76]
Lithography Techniques	top-down	Fe and Fe-based compounds Co and Co-based compounds Ni and Ni-based compounds	Flexible in designing nanoparticles low resolution high cost	[70,77]

## References

[1] Wang Y., Zhao R., Wang S., Liu Z., Tang R. (2016). In vivo Dual-Targeted Chemotherapy of Drug Resistant Cancer by Rationally Designed Nanocarrier. Biomaterials.

[2] Pan Y., Xue P., Liu S., Zhang L., Guan Q., Zhu J., Tian X. (2018). Metal-Based Hybrid Nanoparticles as Radiosensitizers in Cancer Therapy. Colloid Interface Sci. Commun..

[3] Rackwitz T., Debus J. (2019). Clinical Applications of Proton and Carbon Ion Therapy. Semin. Oncol..

[4] Peng F., Su Y., Ji X., Zhong Y., Wei X., He Y. (2014). Doxorubicin-Loaded Silicon Nanowires for the Treatment of Drug-Resistant Cancer Cells. Biomaterials.

[5] Chen X.-Z., Hoop M., Shamsudhin N., Huang T., Özkale B., Li Q., Siringil E., Mushtaq F., Di Tizio L., Nelson B.J. (2017). Hybrid Magnetoelectric Nanowires for Nanorobotic Applications: Fabrication, Magnetoelectric Coupling, and Magnetically Assisted in Vitro Targeted Drug Delivery. Adv. Mater..

[6] Doucey M.-A., Carrara S. (2019). Nanowire Sensors in Cancer. Trends Biotechnol..

[7] Jones R.S., Draheim R.R., Roldo M. (2018). Silver Nanowires: Synthesis, Antibacterial Activity and Biomedical Applications. Appl. Sci..

[8] Wang Z., Wu A., Colombi Ciacchi L., Wei G. (2018). Recent Advances in Nanoporous Membranes for Water Purification. Nanomaterials.

[9] Irshad I., Ahmad F., Mohammed N. (2012). A Review on Nanowires as an Alternative High Density Magnetic Storage Media. AIP Conf. Proc..

[10] Bayrak T., Jagtap N.S., Erbe A. (2018). Review of the Electrical Characterization of Metallic Nanowires on DNA Templates. Int. J. Mol. Sci..

[11] Martínez-Banderas A.I., Aires A., Teran F.J., Perez J.E., Cadenas J.F., Alsharif N., Ravasi T., Cortajarena A.L., Kosel J. (2016). Functionalized Magnetic Nanowires for Chemical and Magneto-Mechanical Induction of Cancer Cell Death. Sci. Rep..

[12] Heidarshenas B., Wei H., Ali Moghimi Z., Hussain G., Baniasadi F., Naghieh G. (2019). Nanowires in Magnetic Drug Targeting. Mater. Sci. Eng. Int. J..

[13] Esteban-Fernández de Ávila B., Ramírez-Herrera D.E., Campuzano S., Angsantikul P., Zhang L., Wang J. (2017). Nanomotor-Enabled Ph-Responsive Intracellular Delivery of Caspase-3: Toward Rapid Cell Apoptosis. ACS Nano.

[14] Assunção-Silva R.C., Gomes E.D., Silva N.A., Salgado A.J., Mozafari M., Rajadas J., Kaplan D. (2019). Chapter 8-Nanoengineered Biomaterials for Spinal Cord Regeneration. Nanoengineered Biomaterials for Regenerative Medicine.

[15] Pondman K., Maijenburg W., Celikkol B., Pathan A.A., Kishore U., Ten Haken B., Ten Elshof A. (2013). Au Coated Ni Nanowires with Tuneable Dimensions for Biomedical Applications. J. Mater. Chem..

[16] Lisjak D., Mertelj A. (2018). Anisotropic Magnetic Nanoparticles: A Review of Their Properties, Syntheses and Potential Applications. Prog. Mater. Sci..

[17] Pondman K.M., Bunt N.D., Maijenburg A.W., van Wezel R.J.A., Kishore U., Abelmann L., ten Elshof J.E., ten Haken B. (2015). Magnetic Drug Delivery with Fepd Nanowires. J. Magn. Magn. Mater..

[18] Banerjee S., Dan A., Chakravorty D. (2002). Review Synthesis of Conducting Nanowires. J. Mater. Sci..

[19] Biswas A., Bayer I.S., Biris A.S., Wang T., Dervishi E., Faupel F. (2012). Advances in Top–Down and Bottom–up Surface Nanofabrication: Techniques, Applications & Future Prospects. Adv. Colloid Interface Sci..

[20] Mu Q., Yan B. (2019). Editorial: Nanoparticles in Cancer Therapy-Novel Concepts, Mechanisms, and Applications. Front. Pharmacol..

[21] Fernandes C., Suares D., Yergeri M.C. (2018). Tumor Microenvironment Targeted Nanotherapy. Front. Pharmacol..

[22] Latorre A., Couleaud P., Aires A., Cortajarena A.L., Somoza Á. (2014). Multifunctionalization of Magnetic Nanoparticles for Controlled Drug Release: A General Approach. Eur. J. Med. Chem..

[23] Hoosen Y., Pradeep P., Kumar P., du Toit L.C., Choonara Y.E., Pillay V. (2018). Nanotechnology and Glycosaminoglycans: Paving the Way Forward for Ovarian Cancer Intervention. Int. J. Mol. Sci..

[24] Qin W., Zheng Y., Qian B.-Z., Zhao M. (2017). Prostate Cancer Stem Cells and Nanotechnology: A Focus on Wnt Signaling. Front. Pharmacol..

[25] Guo D., Ji X., Wang H., Bin S., Chu B., Shi Y., Su Y., He Y. (2018). Silicon Nanowire-Based Multifunctional Platform for Chemo-Photothermal Synergistic Cancer Therapy. J. Mater. Chem. B.

[26] Tan H., Huang Y., Xu J., Chen B., Zhang P., Ye Z., Liang S., Xiao L., Liu Z. (2017). Spider Toxin Peptide Lycosin-I Functionalized Gold Nanoparticles for in Vivo Tumor Targeting and Therapy. Theranostics.

[27] Chen C.-W., Syu W.-J., Huang T.-C., Lee Y.-C., Hsiao J.-K., Huang K.-Y., Yu H.-P., Liao M.-Y., Lai P.-S. (2017). Encapsulation of Au/Fe_3_o_4_ Nanoparticles into a Polymer Nanoarchitecture with Combined near Infrared-Triggered Chemo-Photothermal Therapy Based on Intracellular Secondary Protein Understanding. J. Mater. Chem. B.

[28] Li T., Liu H., Xi G., Pang Y., Wu L., Wang X., Chen T. (2016). One-Step Reduction and Peiylation of Pegylated Nanographene Oxide for Highly Efficient Chemo-Photothermal Therapy. J. Mater. Chem. B.

[29] Sensenig R., Sapir Y., MacDonald C., Cohen S., Polyak B. (2012). Magnetic Nanoparticle-Based Approaches to Locally Target Therapy and Enhance Tissue Regeneration in Vivo. Nanomedicine.

[30] Liu Y.-L., Chen D., Shang P., Yin D.-C. (2019). A Review of Magnet Systems for Targeted Drug Delivery. J. Control. Release.

[31] Shen S., Wu Y., Liu Y., Wu D. (2017). High Drug-Loading Nanomedicines: Progress, Current Status, and Prospects. Int. J. Nanomed..

[32] Alsharif N., Martinez Banderas A., Merzaban J., Ravasi T., Kosel J. (2018). Biofunctionalizing Magnetic Nanowires toward Targeting and Killing Leukemia Cancer Cells. IEEE Trans. Magn..

[33] Zhu H., Deng J., Yang Y., Li Y., Shi J., Zhao J., Deng Y., Chen X., Yang W. (2019). Cobalt Nanowire-Based Multifunctional Platform for Targeted Chemo-Photothermal Synergistic Cancer Therapy. Colloids Surf. B.

[34] Su Y., Wei X., Peng F., Zhong Y., Lu Y., Su S., Xu T., Lee S.-T., He Y. (2012). Gold Nanoparticles-Decorated Silicon Nanowires as Highly Efficient near-Infrared Hyperthermia Agents for Cancer Cells Destruction. Nano Lett..

[35] Egolf P.W., Shamsudhin N., Pané S., Vuarnoz D., Pokki J., Pawlowski A.-G., Tsague P., Marco B., Bovy W., Tucev S. (2016). Hyperthermia with Rotating Magnetic Nanowires Inducing Heat into Tumor by Fluid Friction. J. Appl. Phys..

[36] Lin W.-S., Lin H.-M., Chen H.-H., Hwu Y.-K., Chiou Y.-J. (2013). Shape Effects of Iron Nanowires on Hyperthermia Treatment. J. Nanomater..

[37] Fernandez-Roldan J.A., Serantes D., del Real R.P., Vazquez M., Chubykalo-Fesenko O. (2018). Micromagnetic Evaluation of the Dissipated Heat in Cylindrical Magnetic Nanowires. Appl. Phys. Lett..

[38] Contreras M.F., Zaher A., Perez J.E., Ravasi T., Kosel J. Magnetic Nanowires and Hyperthermia: How Geometry and Material Affect Heat Production Efficiency. Proceedings of the 2015 IEEE International Magnetics Conference (INTERMAG).

[39] Alonso J., Khurshid H., Sankar V., Nemati Z., Phan M.H., Garayo E., García J.A., Srikanth H. (2015). Feco Nanowires with Enhanced Heating Powers and Controllable Dimensions for Magnetic Hyperthermia. J. Appl. Phys..

[40] Spirou S.V., Basini M., Lascialfari A., Sangregorio C., Innocenti C. (2018). Magnetic Hyperthermia and Radiation Therapy: Radiobiological Principles and Current Practice. Nanomaterials.

[41] Choi D.S., Park J., Kim S., Gracias D.H., Cho M.K., Kim Y.K., Fung A., Lee S.E., Chen Y., Khanal S. (2008). Hyperthermia with Magnetic Nanowires for Inactivating Living Cells. J. Nanosci. Nanotechnol..

[42] Hopkins X., Gill W.A., Kringel R., Wang G., Hass J., Acharya S., Park J., Jeon I.T., An B.H., Lee J.S. (2016). Radio Frequency-Mediated Local Thermotherapy for Destruction of Pancreatic Tumors Using Ni–Au Core–Shell Nanowires. Nanotechnology.

[43] Contreras M.F., Sougrat R., Zaher A., Ravasi T., Kosel J. (2015). Non-Chemotoxic Induction of Cancer Cell Death Using Magnetic Nanowires. Int. J. Nanomed..

[44] Fung A., Kapadia V., Pierstorff E., Ho D., Chen Y. (2008). Induction of Cell Death by Magnetic Actuation of Nickel Nanowires Internalized by Fibroblasts. J. Phys. Chem. C.

[45] Zablotskii V., Lunov O., Novotna B., Churpita O., Trosan P., Holan V., Syková E., Dejneka A., Kubinova S. (2014). Down-Regulation of Adipogenesis of Mesenchymal Stem Cells by Oscillating High-Gradient Magnetic Fields and Mechanical Vibration. Appl. Phys. Lett..

[46] Serrà A., Vázquez-Mariño G., García-Torres J., Bosch M., Vallés E. (2018). Magnetic Actuation of Multifunctional Nanorobotic Platforms to Induce Cancer Cell Death. Adv. Biosyst..

[47] Nielsch K., Wehrspohn R., Barthel J., Gosele U., Fischer S., Kronmüller H. (2001). Hexagonally Ordered 100 Nm Period Nickel Nanowire Arrays. Appl. Phys. Lett..

[48] Vega V., Böhnert T., Martens S., Waleczek M., Moreno J., Görlitz D., Prida V., Nielsch K. (2012). Tuning the Magnetic Anisotropy of Coni Nanowires: Comparison between Single Nanowires and Nanowire Arrays in Hard-Anodic Aluminum Oxide Membranes. Nanotechnology.

[49] Ferré R., Ounadjela K., George J.M., Piraux L., Dubois S. (1997). Magnetization Processes in Nickel and Cobalt Electrodeposited Nanowires. Phys. Rev. B.

[50] Hergt R., Dutz S., Müller R., Zeisberger M. (2006). Magnetic Particle Hyperthermia: Nanoparticle Magnetism and Materials Development for Cancer Therapy. J. Phys. Condens. Matter.

[51] Glöckl G., Hergt R., Zeisberger M., Dutz S., Nagel S., Weitschies W. (2006). The Effect of Field Parameters, Nanoparticle Properties and Immobilization on the Specific Heating Power in Magnetic Particle Hyperthermia. J. Phys..

[52] Kossatz S., Grandke J., Couleaud P., Latorre A., Aires A., Crosbie-Staunton K., Ludwig R., Dahring H., Ettelt V., Lazaro-Carrillo A. (2015). Efficient Treatment of Breast Cancer Xenografts with Multifunctionalized Iron Oxide Nanoparticles Combining Magnetic Hyperthermia and Anti-Cancer Drug Delivery. Breast Cancer Res..

[53] Gao W., Kagan D., Pak O.S., Clawson C., Campuzano S., Chuluun-Erdene E., Shipton E., Fullerton E.E., Zhang L., Lauga E. (2012). Cargo-Towing Fuel-Free Magnetic Nanoswimmers for Targeted Drug Delivery. Small.

[54] Gao W., de Ávila B.E.-F., Zhang L., Wang J. (2018). Targeting and Isolation of Cancer Cells Using Micro/Nanomotors. Adv. Drug Deliv. Rev..

[55] Liu X.L., Fan H.M. (2014). Innovative Magnetic Nanoparticle Platform for Magnetic Resonance Imaging and Magnetic Fluid Hyperthermia Applications. Curr. Opin. Chem. Eng..

[56] Bose R.J.C., Lee S.-H., Park H. (2016). Biofunctionalized Nanoparticles: An Emerging Drug Delivery Platform for Various Disease Treatments. Drug Discov. Today.

[57] Ivanov Y.P., Alfadhel A., Alnassar M., Perez J.E., Vazquez M., Chuvilin A., Kosel J. (2016). Tunable Magnetic Nanowires for Biomedical and Harsh Environment Applications. Sci. Rep..

[58] Martinez Banderas A. (2016). A Combined Chemical and Magneto-Mechanical Induction of Cancer Cell Death by the Use of Functionalized Magnetic Iron Nanowires.

[59] Sneider A., VanDyke D., Paliwal S., Rai P. (2017). Remotely Triggered Nano-Theranostics for Cancer Applications. Nanotheranostics.

[60] Lee N., Hyeon T. (2012). Designed Synthesis of Uniformly Sized Iron Oxide Nanoparticles for Efficient Magnetic Resonance Imaging Contrast Agents. Chem. Soc. Rev..

[61] Shore D., Pailloux S.L., Zhang J., Gage T., Flannigan D.J., Garwood M., Pierre V.C., Stadler B.J.H. (2016). Electrodeposited Fe and Fe–Au Nanowires as Mri Contrast Agents. Chem. Commun..

[62] Bañobre-López M., Bran C., Rodríguez-Abreu C., Gallo J., Vázquez M., Rivas J. (2017). A Colloidally Stable Water Dispersion of Ni Nanowires as an Efficient T2-Mri Contrast Agent. J. Mater. Chem. B.

[63] Staňo M., Fruchart O., Brück E. (2018). Chapter 3-Magnetic Nanowires and Nanotubes. Handbook of Magnetic Materials.

[64] Sumanth Kumar D., Jai Kumar B., Mahesh H.M., Mohan Bhagyaraj S., Oluwafemi O.S., Kalarikkal N., Thomas S. (2018). Chapter 3-Quantum Nanostructures (Qds): An Overview. Synthesis of Inorganic Nanomaterials.

[65] Li X., Sun L., Wang H., Xie K., Long Q., Lai X., Liao L. (2016). Synthesis of Cobalt Nanowires in Aqueous Solution under an External Magnetic Field. Beilstein J. Nanotechnol..

[66] Zhao Z.-J., Hwang S.H., Jeon S., Jung J.-Y., Lee J., Choi D.-G., Choi J.-H., Park S.-H., Jeong J.-H. (2017). Effects of Polymer Surface Energy on Morphology and Properties of Silver Nanowire Fabricated Via Nanoimprint and E-Beam Evaporation. Appl. Surf. Sci..

[67] Datta A., Sangle A., Hardingham N., Cooper C., Kraan M., Ritchie D., Narayan V., Kar-Narayan S. (2017). Structure and Thermoelectric Properties of Bi2−Xsbxte3 Nanowires Grown in Flexible Nanoporous Polycarbonate Templates. Materials.

[68] DeMeo D., Macnaughton S., Sonkusale S., Vandervelde T., Hashim A. (2011). Electrodeposited Copper Oxide and Zinc Oxide Core-Shell Nanowire Photovoltaic Cells. Nanowires-Implementations and Applications.

[69] Yu Y., Li J., Wang J., Wu X., Yu C., Xu T., Chang B., Sun H., Arandiyan H. (2019). Orientation Growth and Magnetic Properties of Electrochemical Deposited Nickel Nanowire Arrays. Catalysts.

[70] Serrà A., Vallés E. (2018). Advanced Electrochemical Synthesis of Multicomponent Metallic Nanorods and Nanowires: Fundamentals and Applications. Appl. Mater. Today.

[71] Oviroh P.O., Akbarzadeh R., Pan D., Coetzee R.A.M., Jen T.-C. (2019). New Development of Atomic Layer Deposition: Processes, Methods and Applications. Sci. Technol. Adv. Mater..

[72] Martín-Palma R.J., Lakhtakia A., Lakhtakia A., Martín-Palma R.J. (2013). Chapter 15-Vapor-Deposition Techniques. Engineered Biomimicry.

[73] Parasuraman K., Raghunathan V. (2006). Status of Pulsed Laser Deposition: Challenges and Opportunities. Surf. Eng..

[74] De Teresa J., Fernández-Pacheco A., Córdoba R., Serrano-Ramón L., Sangiao S., Ibarra M. (2016). Review of Magnetic Nanostructures Grown by Focused Electron Beam Induced Deposition (Febid). J. Phys. D.

[75] Nguyen D.M., Bich H.N., Hai Anh P.D., Ai-Le P.H., Bui Q.B. (2019). Vertical Copper Oxide Nanowire Arrays Attached Three-Dimensional Macroporous Framework as a Self-Supported Sensor for Sensitive Hydrogen Peroxide Detection. Arab. J. Chem..

[76] Pirouzfar A., Seyyed Ebrahimi S.A. (2014). Optimization of Sol–Gel Synthesis of Cofe_2_o_4_ Nanowires Using Template Assisted Vacuum Suction Method. J. Magn. Magn. Mater..

[77] Hamdana G., Südkamp T., Descoins M., Mangelinck D., Caccamo L., Bertke M., Wasisto H.S., Bracht H., Peiner E. (2017). Towards Fabrication of 3d Isotopically Modulated Vertical Silicon Nanowires in Selective Areas by Nanosphere Lithography. Microelectron. Eng..

[78] Ferreira J.M., Souza K.P., Queiroz F.M., Costa I., Tomachuk C.R. (2016). Electrochemical and Chemical Characterization of Electrodeposited Zinc Surface Exposed to New Surface Treatments. Surf. Coat. Technol..

[79] Loto C.A. (2016). Electroless Nickel Plating–A Review. Silicon.

[80] Asadian E., Ghalkhani M., Shahrokhian S. (2019). Electrochemical Sensing Based on Carbon Nanoparticles: A Review. Sens. Actuators B.

[81] Jamkhande P.G., Ghule N.W., Bamer A.H., Kalaskar M.G. (2019). Metal Nanoparticles Synthesis: An Overview on Methods of Preparation, Advantages and Disadvantages, and Applications. J. Drug Deliv. Sci. Technol..

[82] Ghosh S. (2019). Electroless Copper Deposition: A Critical Review. Thin Solid Films.

[83] Possin G.E. (1970). A Method for Forming Very Small Diameter Wires. Rev. Sci. Instrum..

[84] Pérez-Page M., Yu E., Li J., Rahman M., Dryden D.M., Vidu R., Stroeve P. (2016). Template-Based Syntheses for Shape Controlled Nanostructures. Adv. Colloid Interface Sci..

[85] Gontad F., Caricato A.P., Cesaria M., Resta V., Taurino A., Colombelli A., Leo C., Klini A., Manousaki A., Convertino A. (2017). Decoration of Silica Nanowires with Gold Nanoparticles through Ultra-Short Pulsed Laser Deposition. Appl. Surf. Sci..

[86] Li H., Guan L., Xu Z., Zhao Y., Sun J., Wu J., Xu N. (2016). Synthesis and Characterization of Amorphous Sio_2_ Nanowires Via Pulsed Laser Deposition Accompanied by N2 Annealing. Appl. Surf. Sci..

[87] Nikov R.G., Dikovska A.O., Atanasova G.B., Avdeev G.V., Nedyalkov N.N. (2018). Magnetic-Field-Assisted Formation of Oriented Nanowires Produced by Pld in Open Air. Appl. Surf. Sci..

[88] Qiu Z., Gong H., Yang X., Zhang Z., Han J., Cao B., Nakamura D., Okada T. (2015). Phosphorus Concentration Dependent Microstructure and Optical Property of Zno Nanowires Grown by High-Pressure Pulsed Laser Deposition. J. Phys. Chem. C.

[89] Shkurmanov A., Sturm C., Hochmuth H., Grundmann M. (2016). Growth Kinetics of Ultrathin Zno Nanowires Grown by Pulsed Laser Deposition. Procedia Eng..

[90] Nikov R.G., Dikovska A.O., Avdeev G.V., Amoruso S., Ausanio G., Nedyalkov N.N. (2019). Pld Fabrication of Oriented Nanowires in Magnetic Field. Appl. Surf. Sci..

[91] Contreras M.F., Ravasi T., Kosel J. Targeted Cancer Cell Death Induced by Induced by Biofunctionalized Magnetic Nanowires. Proceedings of the 2nd Middle East Conference on Biomedical Engineering.

[92] Sun D. (2016). Effect of Zeta Potential and Particle Size on the Stability of Sio_2_ Nanospheres as Carrier for Ultrasound Imaging Contrast Agents. Int. J. Electrochem. Sci..

[93] Hussain M., Xie J., Hou Z., Shezad K., Xu J., Wang K., Gao Y., Shen L., Zhu J. (2017). Regulation of Drug Release by Tuning Surface Textures of Biodegradable Polymer Microparticles. ACS Appl. Mater. Interfaces.

[94] Zhang H., Xu H., Wu M., Yufang Z., Wang D., Jiao Z. (2015). A Soft–Hard Template Approach Towards Hollow Mesoporous Silica Nanoparticles with Rough Surfaces for Controlled Drug Delivery and Protein Adsorption. J. Mater. Chem. B.

[95] Peng F., Su Y., Wei X., Lu Y., Zhou Y., Zhong Y., Lee S.-T., He Y. (2013). Silicon-Nanowire-Based Nanocarriers with Ultrahigh Drug-Loading Capacity for in Vitro and in Vivo Cancer Therapy. Angew. Chem. Int. Ed. Commun..

[96] Wang J., Kumeria T., Bezem M.T., Wang J., Sailor M.J. (2018). Self-Reporting Photoluminescent Porous Silicon Microparticles for Drug Delivery. ACS Appl. Mater. Interfaces.

[97] Lorenz M.R., Holzapfel V., Musyanovych A., Nothelfer K., Walther P., Frank H., Landfester K., Schrezenmeier H., Mailänder V. (2006). Uptake of Functionalized, Fluorescent-Labeled Polymeric Particles in Different Cell Lines and Stem Cells. Biomaterials.

[98] Murugan K., Choonara Y.E., Kumar P., Bijukumar D., du Toit L.C., Pillay V. (2015). Parameters and Characteristics Governing Cellular Internalization and Trans-Barrier Trafficking of Nanostructures. Int. J. Nanomed..

[99] Chithrani B.D., Ghazani A.A., Chan W.C. (2006). Determining the Size and Shape Dependence of Gold Nanoparticle Uptake into Mammalian Cells. Nano Lett..

[100] Frohlich E. (2012). The Role of Surface Charge in Cellular Uptake and Cytotoxicity of Medical Nanoparticles. Int. J. Nanomed..

[101] Behzadi S., Serpooshan V., Hamaly M., Alkawareek M., Dreaden E., Brown D., Alkilany A., Farokhzad O., Mahmoudi M. (2017). Cellular Uptake of Nanoparticles: Journey inside the Cell. Chem. Soc. Rev..

[102] Panzarini E., Mariano S., Carata E., Mura F., Rossi M., Dini L. (2018). Intracellular transport of silver and gold nanoparticles and biological responses: An update. Int. J. Mol. Sci..

[103] Awaad A., Nakamura M., Ishimura K. (2012). Imaging of Size-Dependent Uptake and Identification of Novel Pathways in Mouse Peyer’s Patches Using Fluorescent Organosilica Particles. Nanomedicine.

[104] Prina-Mello A., Diao Z., Coey J.M.D. (2006). Internalization of Ferromagnetic Nanowires by Different Living Cells. J. Nanobiotechnol..

[105] Song M.M., Song W.J., Bi H., Wang J., Wu W.L., Sun J., Yu M. (2010). Cytotoxicity and Cellular Uptake of Iron Nanowires. Biomaterials.

[106] Choi D., Fung A., Moon H., Ho D., Chen Y., Kan E., Rheem Y., Yoo B., Myung N. (2007). Transport of Living Cells with Magnetically Assembled Nanowires. Biomed. Microdevices.

[107] Poland C.A., Byrne F., Cho W.S., Prina-Mello A., Murphy F.A., Davies G.L., Coey J.M., Gounko Y., Duffin R., Volkov Y. (2012). Length-Dependent Pathogenic Effects of Nickel Nanowires in the Lungs and the Peritoneal Cavity. Nanotoxicology.

[108] Wong P.K. (1988). Mutagenicity of Heavy Metals. Bull. Environ. Contam. Toxicol..

[109] Žužek Rožman K., Pečko D., Šturm S., Maver U., Nadrah P., Bele M., Kobe S. (2012). Electrochemical Synthesis and Characterization of Fe70pd30 Nanotubes for Drug-Delivery Applications. Mater. Chem. Phys..

[110] Donaldson K., Tran C.L. (2004). An Introduction to the Short-Term Toxicology of Respirable Industrial Fibres. Mutat. Res. Fundam. Mol. Mech. Mutagenesis.

[111] Magrez A., Kasas S., Salicio V., Pasquier N., Seo J.W., Celio M., Catsicas S., Schwaller B., Forro L. (2006). Cellular Toxicity of Carbon-Based Nanomaterials. Nano Lett..

[112] Rahong S., Yasui T., Kaji N., Baba Y. (2016). Recent Developments in Nanowires for Bio-Applications from Molecular to Cellular Levels. Lab Chip.

[113] Jahangirian H., Kalantari K., Izadiyan Z., Rafiee-Moghaddam R., Shameli K., Webster T.J. (2019). A Review of Small Molecules and Drug Delivery Applications Using Gold and Iron Nanoparticles. Int. J. Nanomed..

[114] Safi M., Yan M., Guedeau-Boudeville M.-A., Conjeaud H., Garnier-Thibaud V., Boggetto N., Baeza-Squiban A., Niedergang F., Averbeck D., Berret J.-F. (2011). Interactions between Magnetic Nanowires and Living Cells: Uptake, Toxicity, and Degradation. ACS Nano.

[115] Perez J.E., Contreras M.F., Vilanova E., Felix L.P., Margineanu M.B., Luongo G., Porter A.E., Dunlop I.E., Ravasi T., Kosel J. (2016). Cytotoxicity and Intracellular Dissolution of Nickel Nanowires. Nanotoxicology.

[116] Liu J., Yu M., Zhou C., Zheng J. (2013). Renal Clearable Inorganic Nanoparticles: A New Frontier of Bionanotechnology. Mater. Today.

